# Certain hormonal profiles of postpartum anestrus jersey crossbred cows treated with controlled internal drug release and ovsynch protocol

**DOI:** 10.14202/vetworld.2016.1102-1106

**Published:** 2016-10-18

**Authors:** Dayanidhi Jena, S. Das, B. K. Patra, S. S. Biswal, D. N. Mohanty, P. Samal

**Affiliations:** 1 Department of Animal Reproduction, Gynaecology and Obstetrics, College of Veterinary Science and Animal Husbandry, Orissa University of Agriculture and Technology, Bhubaneswar - 751 003, Odisha, India; 2 Teaching Veterinary Clinical Complex, College of Veterinary Science and Animal Husbandry, Orissa University of Agriculture and Technology, Bhubaneswar - 751 003, Odisha, India; 3 Department of Clinical Veterinary Medicine, Ethics and Jurisprudence, College of Veterinary Science and Animal Husbandry, Orissa University of Agriculture and Technology, Bhubaneswar - 751 003, Odisha, India

**Keywords:** controlled internal drug release, ovsynch, postpartum anestrus

## Abstract

**Aim:**

The study was conducted to determine the serum levels of certain hormones in post-partum anestrus cows following treatment with controlled internal drug release (CIDR) and Ovsynch protocol.

**Materials and Methods:**

A total of 30 postpartum anestrus cows were divided into three equal groups after thorough gynecoclinical examination. The Group 1 animals received an intravaginal progesterone device on day 0 and 2 ml of prostaglandin F_2α_ (PGF_2α_) on day of CIDR removal (7^th^ day), Group 2 cows were treated with ovsynch protocol (gonadotropin-releasing hormone [GnRH]-PGF_2α_-GnRH) on day 0, 7 and 9, respectively, and Group 3 cows were supplemented with mineral mixture and treated as control. The serum estrogen, progesterone, triiodothyronine, and thyroxine concentration were estimated using enzyme-linked immunosorbent assay kit and absorbance was read at 450 nm with Perkin Elmer Wallac 1420 Microplate Reader.

**Results:**

There was a significant increase in progesterone level in Group 1 after withdrawal of CIDR as compared to other two groups. However, the estrogen assay revealed a greater concentration in Group 2 against Group 1 on day 7 of sampling. However, there was no significant difference for serum triiodothyronine (T_3_) and thyroxine (T_4_) irrespective of treatment protocols and days of sampling.

**Conclusion:**

Treatment with CIDR based progesterone therapy and drug combinations may affect the reproductive hormonal balance like estrogen and progesterone, which is inevitable for successful return to cyclicity and subsequent fertilization and conception. However, as far as serum T_3_ and T_4_ concentration concerned it may not give an astounding result.

## Introduction

Anestrus is a kind of infertility broadly signifying a lack of estrus expression at an expected time. More significantly, it means quiescent, functionless ovaries and reproductive tract. There is expression of transitional change in body physiology brought about by several intrinsic and extrinsic factors with clinical manifestation of lack of estrus. Anestrus is commonly observed either after parturition as postpartum or pre-service anestrus or following service as post service anestrus when conception does not occur. The cow may not come to estrus 30-120 days or longer after parturition but upon rectal examination the cow may show the presence of a normal corpus luteum (CL) suggesting occurrence of ovulation [1]. It may be due to insufficient amount of estradiol secretion which plays a very critical role in the manifestation of the characteristic estrus behaviors.

Progesterone, a key player in the estrus induction, potentiates the action of estrogen which is aided by the regressing CL by secreting some amount of progesterone. Progesterone may decrease the number of hypothalamic estradiol-17 β-receptors and thereby diminishes the potency of estradiol-17 β-negative feedback. The treatment of such cows with progesterone diminish the supportive effect of estradiol 17 β, enabling sufficient luteinizing hormone (LH) secretion to stimulate preovulatory follicular development [2] and the mechanisms of controlling the formation and involution of CL. It has been suggested that greater blood progesterone concentration during luteal phase preceding insemination increases conception rate in dairy cows and greater progesterone level before insemination reduces uterine secretion of prostaglandin F_2α_ (PGF_2α_) (in response to oxytocin) during the late luteal phase after insemination [3]. Exogenous progestins are considered appropriate for noncyclic anestrus postpartum cows [4]. When exogenous progesterone is used in the synchronization protocol, a desirable follicle is produced and been shown to elicit an increase in LH pulse frequency in postpartum anestrus cows [5]. The progesterone from the controlled internal drug release (CIDR) was sufficient to increase and maintain a progesterone concentration in blood >2 ng/ml in the absence of CL on the ovary. Blood progesterone level rises rapidly after insertion of CIDR and declined rapidly within 24 h after its removal causing ovulatory estrus [4,6].

The triiodothyronine (T_3_) and thyroxin (T_4_) composed of iodinated amino acids, under the regulation of thyrotropic or thyroid stimulating hormone are secreted from thyroid gland. Thyroid gland activity is having utmost importance on the reproductive performance of the animal which may be directly or indirectly associated with infertility. The objective of the study was to assess the serum profile of estrogen, progesterone, triiodothyronine, and thyroxine in the experimental animals.

## Materials and Methods

### Ethical approval

The experimental procedures have been conducted in accordance with the guidelines laid down by the Institutional Ethics Committee.

### Study site

The present investigation was undertaken in the Department of Animal Reproduction, Gynecology and Obstetrics, College of Veterinary Science and Animal Husbandry, Orissa University of Agriculture and Technology, Bhubaneswar, Odisha, situated at a geographical coordination of 20.2961°N, 85.8245°E.

### Source of animal

The experiment was conducted in postpartum anestrus crossbred cows, presented in the Teaching Veterinary Clinical Complex, College of Veterinary Science and Animal Husbandry, Orissa University of Agriculture and Technology, Bhubaneswar and cases attended at mobile health coverage scheme, OUAT at owner’s residence in and around Bhubaneswar city. Those cows were owned by private cow owners and maintained by traditional husbandry practices. Lactating cows from 1^st^ to 5^th^ calver not exhibiting estrus for 90 days or more postpartum were selected for this study. Apparently, healthy cows not suffering from intercurrent and metabolic diseases were included.

### Sample collection

Blood samples were collected from jugular venipuncture of selected animals during the early morning on day 0, 7, and at the time of artificial insemination (AI) of the experiment. About 5 ml of blood was collected from each selected animal and serum was separated by centrifuging it. The clear serum was harvested, kept in dry sterile cryovials and stored in deep freeze at −20°C for further estimation.

### Hormonal analysis

Serum estrogen concentration was evaluated on the principle of competitive binding between estrogen in the test specimen and estrogen enzyme conjugate for a constant amount of anti-estradiol polyclonal antibody using enzyme-linked immunosorbent assay (ELISA) kit. The serum progesterone and T_3_ concentration were evaluated using solid phase competitive ELISA. The serum T_4_ concentration was evaluated by competitive ELISA using a streptavidin coated assay plate of ELISA T_4_ kit. Absorbance was read at 450 nm with a microplate reader (WALLAC 1420, Multilabel counter, Perkin Elmer life and Analytical Sciences) within 15 min. The mean absorbance value (A_450_) for each set of reference standards, control, and samples was calculated.

### Treatment protocol

About 30 postpartum anestrus crossbred Jersy cows were selected following gynecoclinical examination and divided into three equal groups. Those animals selected were allotted randomly into three groups.

#### Group 1 (n=10)

This group of animals was treated with CIDR on day 0 of experiment and kept *in situ* for 7 days followed by 2 ml of PGF_2α_ through intramuscular route on the day of removal of CIDR. Fixed time insemination was done in cows at induced estrus after 48 h.

#### Group 2

This group of animals was treated with ovsynch protocols (gonadotropin-releasing hormone [GnRH]-PGF_2α_-GnRH combination). Accordingly, these animals received 5 ml of a GnRH analog on day 0 intramuscularly, followed by 2 ml of a PGF_2α_ analog intramuscularly on day 7 and again 5 ml of GnRH I/M on day 9. Fixed-time AI was done at 24 h after the second dose of GnRH injection.

#### Group 3

This group of animals received only mineral mixture 100 g daily for 15 days and considered as control for comparison study.

### Statistical analysis

Data generated were subjected to statistical analysis by one-way analysis of variance using statistical package for social sciences (SPSS; version 22). Difference between mean was tested using Duncan’s multiple comparison test. Results were expressed as mean±standard error.

## Results

### Serum estrogen concentration

The serum estrogen concentrations on day 0, 7 and at the time of AI in different experimental groups were tabulated in Table-1. In the Group 1 (CIDR+PGF_2α_), the serum estrogen values (pg/ml) on day 0, 7 and at the time of AI were found to be 41.81±4.21, 30.42±1.61 and 50.00±2.11, respectively, showing significantly lower (p<0.01) value on the 7^th^ day sampling from day 0 and at the time of AI. However, the estrogen value on day of AI did not differed significantly either on day 0 or day 7. In Group 2, the mean value of estrogen at the time of AI was significantly higher (p<0.05) compared to 0 or 7^th^ day observation where the estrogen concentration registered 41.80±5.0, 43.06±2.34 and 52.81±2.5 for different days of sampling.

**Table-1:** Mean serum estrogen concentration (pg/ml) of different experimental groups on different days of sampling in postpartum anestrus cows.

Experimental groups	Days of sampling			F

	0	7	At time of AI	
Group 1: CIDR+PGF_2α_ (10)	41.81±4.21_×z_	30.42±1.61^a^_y_	50.00±2.11^ab^_z_ (10)	14.87**
Group 2: Ovsynch (10)	41.80±5.00_x_	43.06±2.34^b^_x_	52.81±2.50^a^_y_ (7)	3.26*
Group 3: Control (10)	48.94±1.13	-	43.31±4.28^b^ (3)	3.58^NS^
F	1.784^NS^	20.96**	2.36*	-

* p<0.05,

** p<0.01.

Figures in parenthesis indicate number of animals, Figures bearing same superscript in a column do not differ significantly and figures bearing same subscripts in a row do not differ significantly. NS=Non-significant, CIDR=Controlled internal drug release, PGF_2α_=Prostaglandin F_2α_, AI=Artificial insemination

The mean estrogen concentration was 48.94±1.13 pg/ml on day 0 of estimation and did not reveal any difference on day of AI (43.31±4.28) in control group (Group 3) where the cows were fed only with mineral mixture.

The experimental groups did not differ among themselves on day 0 while other two samplings on day 7 and at the time of AI showed variation (p<0.01) in estrogen concentration. The ovsynch treated group showed significantly higher (p<0.05) estrogen value compared to Group 1. Similarly, highest estrogen concentration (p<0.05) was recorded in ovsynch protocol on day of AI than that of control group. However, no such significance in estrogen concentration was observed between Groups 1 and 2 on day of AI.

### Serum progesterone concentration

The serum progesterone concentrations (ng/ml) on day 0, 7 and at the time of AI in different experimental groups were tabulated in Table-2. The pre-treatment progesterone value on day 0 among various experimental groups did not record any significant difference. The CIDR+PGF_2α_ treated cows (Group 1) showed significantly higher (p<0.05) progesterone value (3.57±0.39) on the 7^th^ day compared to Group 2 (1.89±1.00) which was subjected to ovsynch protocol. However, a nonsignificant progesterone value observed on the day of AI averaged between 0.88±0.03 (Group 3) and 1.12±0.22 in Group 2 cows.

**Table-2:** Mean serum progesterone concentration (ng/ml) of different experimental groups on different days of sampling in postpartum anestrus cows.

Experimental groups	Days of sampling			F

	0	7	At time AI	
Group 1: CIDR+PGF_2α_ (10)	1.74±0.34_x_	3.57±0.39_y_^b^	1.02±0.07_x_ (10)	9.81*
Group 2: Ovsynch (10)	1.58±0.45	1.89±1.00^a^	1.12±0.22 (7)	0.614^NS^
Group 3: Control (10)	1.02±0.03	-	0.88±0.03 (3)	4.296^NS^
F	1.859^NS^	3.315*	0.428^NS^	

* p<0.05,

Figures in parenthesis indicate number of animals, Figures bearing same superscript in column do not differ significantly, Figures bearing same subscripts in row do not differ significantly. CIDR=Controlled internal drug release, PGF_2α_=Prostaglandin F_2α_, NS=Non-significant, AI=Artificial insemination

Days of sampling revealed a significant variation (p<0.05) in group 1 in different days of sampling. A higher serum progesterone concentration (3.57±0.39) was recorded on day 7 (p<0.05) in comparison to pre-treatment and at the time of AI where minimum of 1.02±0.07 was observed without any statistical variation between days of sampling in Group 1. On the contrary, progesterone concentration did not differ significantly within days of sampling for remaining experimental groups.

### Serum triiodothyronine and thyroxine concentration

The mean triiodothyronine (T_3_) and thyroxine (T_4_) concentration were depicted in Table-3. The serum triiodothyronine (T_3_) concentration (ng/dl) of Group 1 varied from 0.25±0.04 (at the time of AI) to 0.31±0.10 (day 0). The cows treated with ovsynch protocol (Group 2) registered a value of 0.26±0.03, 0.27±0.05 and 0.40±0.08, respectively, on different days of sampling. In control group of animals, the T_3_ values were 0.41±0.04 and 0.43±0.01, respectively, on day 0 and at the time of AI. No significant difference could be observed within groups or between groups with regard to serum T_3_ concentration.

**Table-3:** Mean serum triiodothyronine (T_3_) and thyroxine (T_4_) concentration of various experimental groups on different days of sampling in postpartum anestrus cows.

Experimental groups	T_3_ (ng/ml) Days of sampling			T_4_ (µg/dl) Days of sampling		
	
	0	7	At time of AI	0	7	At time of AI
Group 1: CIDR+PGF_2α_ (10)	0.31±0.10	0.31±0.05	0.25±0.04 (10)	5.48±0.09	5.55±0.10	5.48±0.07 (10)
Group 2: Ovsynch (10)	0.26±0.03	0.27±0.05	0.40±0.08 (7)	5.54±0.09	5.36±0.08	5.68±0.07 (7)
Group 3: Control (10)	0.41±0.04	-	0.43±0.01 (3)	5.22±0.70	-	5.41±0.16 (3)
F	1.239^NS^	0.334^NS^	1.985^NS^	4.09^NS^	1.860^NS^	2.35^NS^

Figures in parenthesis indicate number of animals. NS=Non-significant, CIDR=Controlled internal drug release, PGF_2α_=Prostaglandin F_2α_, AI=Artificial insemination

The serum thyroxine (T_4_) assay (µg/dl) revealed a value ranged from 5.22±0.70 in Group 3 on day 0 to a maximum concentration of 5.55±0.10 on day 7 sampling in Group 1 cows which did not register any significant difference within days or among groups.

## Discussion

### Serum estrogen

It is an established fact that manifestation of estrus is the principal function of estrogen which acts on the tubular genital tract for its regular functionality and it also sensitizes the central nervous system for coordinating sexual behavior and receptivity to the male animals [1,7]. In the present experiment, estrogen concentration for Group 1 ranged between 41.81±4.21 and 50.00±2.11 and that for Group 2 the values were 41.80±5.00 and 52.81±2.50 which was higher than the earlier observation, where the estradiol concentrated was in a range of 24.38±3.76 to 21.79±1.61 in different days of estrus cycle [4]. With reference to Table-1, there was a significant difference (p<0.05) in serum estrogen level of Group 2 (43.06±2.34) as compared to Group 1 (30.42±1.61) on day 7 of sampling which could be due to the negative effect of progesterone on estrogen in CIDR-treated animals [7,8].

### Serum progesterone

In the present experiment, the progesterone value for Group 1 ranged between 1.02±0.7 to 3.57±0.39; 1.12±0.22 to 1.89±1.00 for Group 2 and 0.88±0.03 to 1.02±0.03 in the case of control group irrespective of days of sampling. The circulating progesterone value in Group 1 increased significantly (p<0.05) to 3.57±0.39 on day of removal than the pre-treatment value of 1.74±0.34 and it was significantly higher (p<0.05) from the Group 2. This finding corroborates the observation made by earlier workers [4,8-11]. However, Mishra *et al*. [12] and Mohapatra *et al*. [13] observed the progesterone concentration from 2.5 to 3.6 ng/ml in postpartum anestrus cows. A significant increase in progesterone concentration in progesterone implanted cows (CIDR+PGF_2α_) might be due to exogenous progesterone and uniform and sustained release of hormone to the vascular system. Hence, the rise of progesterone on the 7^th^ day is obvious due to the effect of CIDR [3].

On the contrary, the serum progesterone value did not differ significantly in the ovsynch group irrespective of days of sampling [5].

### Serum triiodothyronine (T_3_) and thyroxine (T_4_)

The physiological range of T_3_ and T_4_ were 0.41-1.69 (ng/ml) and 4.19-8.60 (µg/dl), respectively. Both the serum T_3_ and T_4_ values did not reveal any significant difference within various experimental groups irrespective of different hormonal treatment and days of sampling. In the present experiment, the serum T_3_ values ranged between 0.25±0.04 and 0.43±0.01 among three groups. Similarly, the mean values for T_4_ were in a range of 5.22±0.70 to 5.68±0.07, respectively. However, the present values were higher from earlier works [3,14].

Similar observation was also noted by earlier workers who reported nonsignificantly lower values for T_3_ and T_4_ hormones in anestrus cows as compared to cyclic cows [15,16]. Hence, it could be concluded that T_3_ and T_4_ hormones may not interfere directly with reproductive functions unless there is systemic influence.

The present observation of T_3_ and T_4_ values in different experimental groups did not find any appreciable changes in the level of aforesaid hormones in anestrus cows [1].

## Conclusion

The study revealed that there was a marked increase in progesterone value on the day of removal of CIDR and the value of progesterone was significantly different between other groups and also within different days of sampling. However, the estrogen concentration was significantly higher in ovsynch treated groups as compared to CIDR group which might be due to the negative effect of the progesterone on estrogen. And as far as serum triiodothyronine and thyroxine is concerned treatment with CIDR or ovsynch has no effect on their values. Further studies need to be done in more and more animals to validate this study.

## Authors’ Contributions

The study is a thesis part of M.V.Sc. degree of DJ. SD, BKP and DNM planned the study and DJ did the research under the guidance of SD. SSB and PS guided in statistical analysis. All authors participated in draft and revision of the manuscript. All authors read and approved the final manuscript.
